# Limited Antineutrophil Cytoplasmic Antibody-Associated Vasculitis Presenting With Diffuse Alveolar Hemorrhage: A Case Report and Literature Review

**DOI:** 10.7759/cureus.62759

**Published:** 2024-06-20

**Authors:** Hema Kondakindi, Joud Enabi, Kejal Shah, Duy Chung, Luan Ngo, Srikanth Mukkera

**Affiliations:** 1 Internal Medicine, Texas Tech University Health Sciences Center, Odessa, USA; 2 Internal Medicine, Northwest Medical Center, Tucson, USA; 3 Internal Medicine, Prasad Medical Center, Brooklyn, USA; 4 Cardiovascular Research, Methodist Hospital, Indiana, USA; 5 Internal Medicine, University at Buffalo Catholic Health, New York City, USA; 6 Rheumatology, Texas Tech University Health Science Center at Permian Basin, Odessa, USA

**Keywords:** vasculitis, case report, rheumatology, alveolar hemorrhage, anca vasculitis

## Abstract

Antineutrophil cytoplasmic antibody (ANCA)-associated vasculitis (AAV) presents a significant medical challenge, with an annual incidence of 2.4 to 4.1 cases per 100,000 individuals and a prevalence of 29.6-54.6 cases per 100,000 individuals in the US. Diffuse alveolar hemorrhage (DAH) is a rare but grave complication of AAV, often associated with a mortality rate as high as 58.3%. This case study highlights a 52-year-old male patient who presented with hemoptysis and was diagnosed with DAH on bronchoscopy, subsequently testing positive for ANCA-PR3. Prompt intervention, including pulse steroids, rituximab, and plasmapheresis, along with the novel FDA-approved drug avacopan, led to significant improvement within four weeks. Early recognition and aggressive management are crucial in mitigating the life-threatening consequences of DAH in AAV, emphasizing the importance of bronchoscopy and advanced therapeutic modalities. This case underscores the potential efficacy of avacopan in managing ANCA vasculitis post-acute phase, offering hope for improved outcomes in this challenging condition.

## Introduction

Antineutrophil cytoplasmic antibody (ANCA)-associated vasculitis (AAV) encompasses microscopic polyangiitis (MPA), granulomatosis with polyangiitis (GPA), and eosinophilic granulomatosis with polyangiitis (EGPA) [1,2]. AAV affects small vessels and is associated with ANCA specific for myeloperoxidase (MPO-ANCA) or proteinase 3 (PR3-ANCA). In the US, AAV has an estimated annual incidence of 2.4-4 cases per 100,000 individuals and a prevalence of 29.6-54 cases per 100,000 individuals [1-5]. Diffuse alveolar hemorrhage (DAH) is a rare but life-threatening AAV complication. Bronchoscopy with bronchoalveolar lavage (BAL) is usually required to confirm the diagnosis. We present a case of a 52-year-old man with DAH confirmed by bronchoscopy and positive ANCA-PR3 [6,7]. The patient showed significant improvement with rituximab and pulse glucocorticoids, followed by discharge on methotrexate and newly FDA-approved avacopan.

## Case presentation

A 52-year-old Hispanic man presented to the emergency room with gradual-onset shortness of breath over the past five to six weeks. His symptoms progressively worsened, were present at rest, and were aggravated with exertion, without alleviating. He also experienced a productive cough two weeks before admission with occasional hemoptysis, low-grade fever, night sweats, weight loss, and poor appetite, which led to his inability to work and be housebound. His medical history was unremarkable, except for frequent traveling between Texas and Mexico and a childhood history of tuberculosis exposure from his father who later died from the disease. The patient denied other signs and symptoms. External-facility CT chest revealed chronic lung disease, bilateral scarring, bronchiectasis, a 5.7 cm soft tissue mass in the posteromedial left lower lobe, and scattered soft tissue nodules. Blood tests from the external facilities, including CBC, CMP, and urine analysis, were unremarkable.

**Figure 1:**
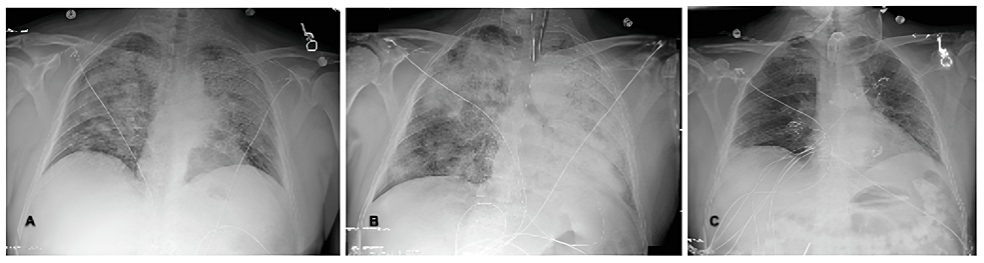
Chest X-ray (CXR) progress over the course of treatment A) CXR on admission showed bilateral multifocal infiltrates with the largest consolidation area in the left upper lobe; B) CXR after intubation showed hypo-inflated lungs and extensive bilateral perihilar infiltrates. C) CXR on discharge showed improvement compared to the previous CXR.

Upon admission, the patient’s condition rapidly deteriorated with severe respiratory distress, hypertension, tachycardia, tachypnea, hypoxia (saturating at 85% on room air), and no fever. On auscultation, bilateral diffuse crackles were heard. Chest X-ray (CXR) revealed bilateral multifocal infiltrates. Clinical presentation and imaging were suggestive of multifocal community-acquired pneumonia (Figure 1A). The patient was admitted to the general medical unit and started on intravenous ceftriaxone and azithromycin.

**Figure 2:**
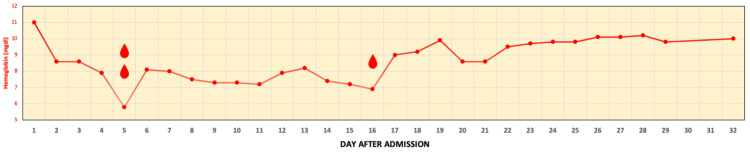
Hemoglobin levels progress over the course of treatment The patient’s hospital stay was significant for the fluctuation of hemoglobin levels and packed red blood cell transfusion indicated by the red droplet symbol.

The patient decompensated with a drop in hemoglobin (Figure 2) and increased oxygen support, requiring a high-flow nasal cannula (HFNC). On day 4, he experienced bouts of cough leading to severe hypoxia with an oxygen saturation of 81% on HFNC, necessitating a transfer to the ICU.

**Figure 3:**
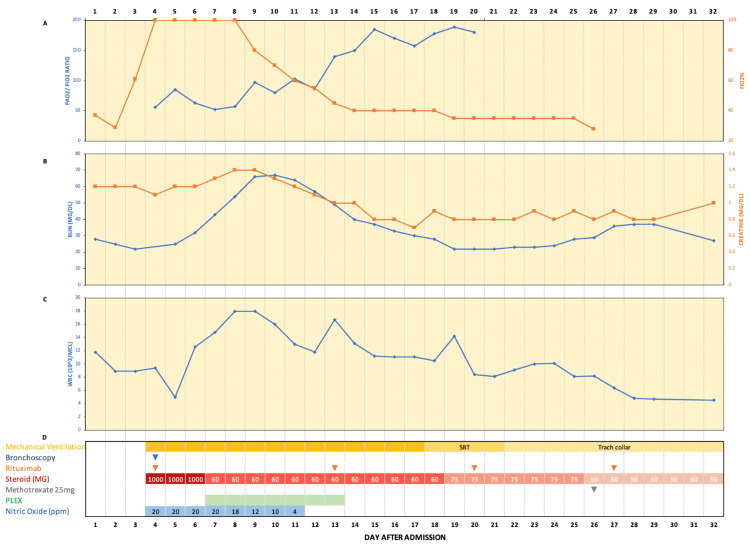
The progress of pulmonary function, kidney function, and white blood cells following the treatments A) The progress of the PaO_2_/FiO_2_ ratio and FiO_2_ over the course of the treatment. B) The progress of BUN and creatinine levels over the course of the treatment. C) The progress of white blood cells over the course of the treatment. D) The treatment plan summary. PaO_2_: partial pressure of oxygen; FiO_2_: fraction of inspired oxygen

The patient developed severe respiratory distress with tachypnea requiring 100% FiO_2_ on HFNC. Noninvasive positive pressure ventilation (BiPAP) was initiated alongside dexmedetomidine to control tachypnea. Due to persistent and worsening hypoxia, endotracheal intubation and mechanical ventilation were required. Post-intubation bronchoscopy showed diffusely erythematous bronchial mucosa and copious bloody secretions in bilateral main bronchi and distal airways, which were partially obstructive without endobronchial lesions. BAL was performed for cytology evaluation and culture. ESR was elevated, suggesting autoimmune or infectious etiology. However, negative pan-cultures made pneumonia less likely. Methylprednisolone pulse glucocorticoid therapy and rituximab were initiated (Figure 3). Nephrology and rheumatology were consulted due to suspected severe DAH secondary to AAV. Nitric oxide was initiated to manage refractory respiratory failure (Figure 3). The antibiotic regimen was adjusted to meropenem, linezolid, levofloxacin, and fluconazole. Despite interventions, his condition remained unchanged with hemodynamic instability and fluctuating hemoglobin levels, necessitating intermittent pressure use and packed red blood cell transfusions (Figure 2). Attempts to wean off nitric oxide were unsuccessful due to persistent hypoxemia. The ANCA panel was equivocal, showing negative indirect immunofluorescence (IIF) but positive enzyme-linked immunoassay (ELISA) immunoglobulin G (IgG) PR3 (Table 1). Pulse dose steroid completion was followed by a tapering dose of steroids after 10 days and 7 days of plasmapheresis. Following treatment, the patient improved after 25 days, evidenced by decreased oxygen requirements with improvement in FiO_2_ and PaO_2_/FiO_2_ (Figure 3A) and a successful attempt to wean down nitric oxide. Positive PR3-ANCA, clinical presentation of DAH, and response to appropriate treatment supported the AAV diagnosis (Table 1). BAL cytology revealed respiratory epithelial cells, histiocytes, and mixed inflammation in the blood background, confirming the diagnosis of DAH.

**Table 1:** Laboratory and culture results

Laboratory	Result
Marker	
Anti-Smith	Negative
Anti-fibrillin	Negative
Anti-GBM	Negative
ANA	Negative
Anti-phospholipid	Negative
dsDNA	Negative
ANCA	
ELISA	PR-3 IgG 983
IIF	Negative
Complement C3	159 mg/dL
Complement C4	30 mg/dL
ESR	>100 mm/h
CRP	24.6 mg/L
Culture	
Blood Culture	Negative
Sputum Culture	Negative
Bronchial Lavage Culture	Streptococcus salivarius Streptococcus anginosus Eggerthella lenta
Legionella	Negative
Strep	Negative
Histoplasma	Negative
TB QuantiFERON	Negative

**Figure 4:**
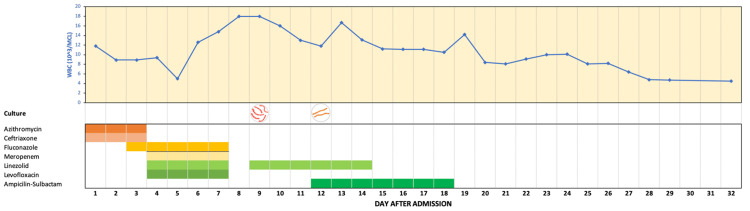
The progress of antibiotics therapy, bacteria cultures, and white blood cells

Given the definitive DAH-AAV diagnosis and preliminary negative pan-cultures, we discontinued empiric broad-spectrum antibiotics. However, linezolid and ampicillin-sulbactam were restarted due to positive BAL fluid cultures for Streptococcus pneumoniae, resulting in the gradual normalization of white blood cells (WBC) (Table 1; Figure 4). The patient remained stable and had an improvement in mental status, allowing a gradual reduction of sedation. Following the completion of plasmapheresis and the PaO_2_/FiO_2_ ratio improvement (Figure 3A), he was weaned from mechanical ventilation and transitioned to tracheostomy ventilation on day 22. He was discharged on the thirty-first day to Inpatient Rehabilitation and treatment was continued with subcutaneous methotrexate, oral prednisone, and oral tavenos. He received a close outpatient follow-up with a rheumatologist. One-month follow-up showed complete disease resolution.

## Discussion

AAV affects small vessels and presents with three syndromes: GPA, MPA, and EGPA. Lung involvement is commonly observed in GPA (associated with anti-PR3 antibodies) and MPA (associated with anti-MPO antibodies). DAH is an uncommon but critical complication of AAV requiring urgent medical attention. In a study by Yi Lin et al. (1994-2007), out of 131 cases of AAV, 12 developed DAH [4]. In nine studies between 1985 and 2012, the DAH incidence in AAV was between 8% and 36% [4]. DAH can be caused by various pathologies, including autoimmune disease (AAV, systemic lupus erythematosus, etc.), infection, and non-immune causes (acute respiratory distress syndrome, bleeding disorders, malignancy, etc.). The clinical presentation of DAH is nonspecific, with dyspnea being the most consistent presenting symptom. Hemoptysis may only occur in one-third of patients, even in severe cases [8,9]. CXR is a nonspecific but highly sensitive imaging modality, with 94% of patients showing radiological evidence of DAH [10]. CXR may reveal new patchy or diffuse alveolar opacities or reticular interstitial opacities with honeycombing (pulmonary fibrosis due to recurrent pulmonary hemorrhage). An abnormal CXR prompts a CT scan, which can range from localized ground-glass opacification to extensive consolidation opacities with air bronchograms [5,11]. The diagnosis of DAH is challenging. Clinicians should raise suspicion for DAH in patients with low hemoglobin, new and severe hypoxic respiratory failure with diffuse alveolar infiltrate, ground glass, or consolidative opacities on imaging, even without hemoptysis. When the diagnosis is in doubt, fiberoptic bronchoscopy and BAL should be considered to look for frank blood, fibrin, or hemosiderin-laden macrophages on cytology to confirm DAH and exclude infection. Increased ESR or CRP is nonspecific and present in infection and vasculitis conditions. A serum autoimmune panel should be obtained when DAH is suspected. ANCA screening includes indirect immunofluorescence (IIF) (detect antibodies) and ELISA (detect MPO and PR3 antigens). Following the 2017 International Consensus initiative in the ANCA field, ELISA is the preferred screening methodology for AAV diagnosis. In our case, the patient developed shortness of breath with low-grade temperature without indicators of systemic disease, which suggested an infection process. However, the patient developed multifocal bilateral lung infiltrates that did not improve with antibiotics along with a sudden drop in hemoglobin. CXR revealed chronic lung disease with scattered soft tissue nodules. These features supported an alternative etiology. Prompt bronchoscopy revealed DAH and serum laboratory results confirmed AAV diagnosis.

Classically, GPA has a multisystem effect with necrotizing granulomatous vasculitis of the respiratory tract, focal necrotizing glomerulonephritis, and generalized focal necrotizing vasculitis. Demonstration of one or more of these combined with positive serological evidence is now considered fulfilling the criteria [12,13]. Limited Wegener’s granulomatosis is characterized by the absence of kidney disease and systemic vasculitis evidence, with a c-ANCA sensitivity of 65-70% [14]. Clinical presentation varies from asymptomatic to cough, dyspnea, hemoptysis, pleuritic chest pain, malaise, weight loss, and fever [15,16]. In 1966, Carrington and Liebow described 16 patients with pulmonary lesions identical to Wegener’s granulomatosis with the absence of or limited lesions elsewhere [17]. In this case, the patient presented with symptoms limited to the respiratory system without systemic vasculitis. The kidney function tests briefly changed, possibly resulting from pre-renal acute kidney injury due to volume depletion secondary to DAH.

DAH-AAV has a high mortality rate of 58.3% [17], emphasizing the need for prompt diagnosis and treatment. Vasculitis therapy involves early disease identification followed by rapid initiation of immunosuppression, consisting of two phases: the “remission-induction” phase to control active disease and the “maintenance” phase to sustain remission with less aggressive treatment. Corticosteroids and immunosuppressive agents remain the gold standard. Before initiating immunosuppression, careful evaluation to exclude infection is required before immunosuppressive therapy. Empiric broad-spectrum antibiotics are often initiated alongside standard therapy while waiting for microbial study results. The selection of a specific immunosuppressive agent for DAH-AVV depends on the severity of disease activity. European Vasculitis Society (EUVAS) proposed grading the vasculitis severity by measuring the number of organ systems involved, the degree of renal disease, and the presence of DAH [17]. It is important to note that DAH is an immediate threat, making DAH-AAV a severe vasculitis presentation. In this case, negative pan-cultures and normal WBC made infection less likely. The patient exhibited severe clinical features with threatened lung function and extreme hypoxic respiratory failure. Combination therapy with corticosteroids, rituximab, and plasma exchange was the preferred treatment for AAV with DAH complications in an acute setting.

Avacopan, a newly FDA-approved drug (2021), has revolutionized the management of ANCA vasculitis. It acts as a complement 5a receptor (C5aR) antagonist and effectively inhibits C5a-mediated neutrophil activation and migration. In the ADVOCATE trial, avacopan demonstrated non-inferiority to glucocorticoids for the AAV treatment but significantly reduced the risk of relapse or sustained remission, preserved kidney function, improved quality of life, and a lower rate of adverse events compared to standard therapy [17].

## Conclusions

AAV is a rare condition but could lead to severe complications, including DAH with high mortality. Given the rarity and insidious clinical presentation of DAH, early identification and a high index of suspicion are necessary in patients with diffuse alveolar infiltrates. The presentation may be limited to a single organ, making diagnostic processes more ambiguous. In cases suspected of AAV with lung involvement, bronchoscopy and BAL should be considered, along with prompt ANCA testing to guide treatment, with ELISA being the preferred test. Treatment should be tailored based on the severity of the vasculitis activity proposed by EUVAS guidelines.
